# RNAInter in 2020: RNA interactome repository with increased coverage and annotation

**DOI:** 10.1093/nar/gkz804

**Published:** 2019-09-17

**Authors:** Yunqing Lin, Tianyuan Liu, Tianyu Cui, Zhao Wang, Yuncong Zhang, Puwen Tan, Yan Huang, Jia Yu, Dong Wang

**Affiliations:** 1 Department of Bioinformatics, School of Basic Medical Sciences, Southern Medical University, Guangzhou 510515, China; 2 College of Bioinformatics Science and Technology, Harbin Medical University, Harbin 150081, China; 3 Shunde Hospital, Southern Medical University (The First People's Hospital of Shunde), Foshan 528308, China; 4 State Key Laboratory of Medical Molecular Biology, Department of Biochemistry & Molecular Biology, Institute of Basic Medical Sciences, Chinese Academy of Medical Sciences (CAMS) & Peking Union Medical College (PUMC), Beijing 100730, China; 5 Dermatology Hospital, Southern Medical University, Guangzhou 510091, China; 6 Center for Informational Biology, University of Electronic Science and Technology of China, Chengdu 611731, China

## Abstract

Research on RNA-associated interactions has exploded in recent years, and increasing numbers of studies are not limited to RNA–RNA and RNA–protein interactions but also include RNA–DNA/compound interactions. To facilitate the development of the interactome and promote understanding of the biological functions and molecular mechanisms of RNA, we updated RAID v2.0 to RNAInter (RNA Interactome Database), a repository for RNA-associated interactions that is freely accessible at http://www.rna-society.org/rnainter/ or http://www.rna-society.org/raid/. Compared to RAID v2.0, new features in RNAInter include (i) 8-fold more interaction data and 94 additional species; (ii) more definite annotations organized, including RNA editing/localization/modification/structure and homology interaction; (iii) advanced functions including fuzzy/batch search, interaction network and RNA dynamic expression and (iv) four embedded RNA interactome tools: RIscoper, IntaRNA, PRIdictor and DeepBind. Consequently, RNAInter contains >41 million RNA-associated interaction entries, involving more than 450 thousand unique molecules, including RNA, protein, DNA and compound. Overall, RNAInter provides a comprehensive RNA interactome resource for researchers and paves the way to investigate the regulatory landscape of cellular RNAs.

## INTRODUCTION

RNA-associated interactions involve many physiological and pathological processes, such as cell growth and development, cell differentiation and inflammation (1–4). With the rapid development of biotechnology techniques, new RNA-associated interactions are being discovering continuously. These new techniques include Degradome-seq (5), LIGR-seq (6), MARIO (7) and PARIS (8) for the detection of RNA–RNA interactions (RRIs); dCLIP (9), PAR-CLIP (10), RIP-seq (11) and uvCLAP (12) for the detection of RNA–protein interactions (RPIs) and ChIRP-seq (13), ChOP-seq (14), diMARGI (15) and GRO-seq (16) for the detection of RNA–DNA interactions (RDIs) (see description in Supplementary Table S1). Recently, the regulatory roles of drug-associated miRNAs and lncRNAs in drug resistance have been a research focus (17–19). Transcription factors (TFs) and histone modifications contribute to the transcriptional regulation of RNA, which participates in various biological processes (20,21). The integration of these is therefore a prerequisite for RNA-related biomarker or mechanistic studies. However, many databases have manually collected and identified RNA-associated interactions through experimental validation and computational prediction from the literature and high-throughput sequencing. The majority of these resources focus on certain types of interactions with insufficient molecular information. Thus, numbers of annotations about RNA and other interactors, such as target sites, RNA editing and RNA modification, should be included. Currently, a global view of the RNA interactome with comprehensive annotations is not available across most species.

**Figure 1.**
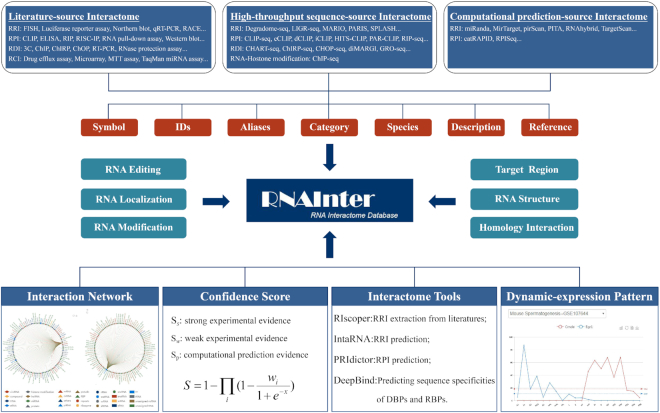
Overview of the RNAInter database.

Here, we updated RAID v2.0 (22) to RNAInter (RNA Interactome Database, http://www.rna-society.org/rnainter/ or http://www.rna-society.org/raid/) to address these challenges. RNAInter establishes a repository of integrated experimentally validated and computationally predicted RNA-associated interactions through manual curation of the literature, along with another 35 resources under one common framework (Figure 1, Table 1). It also supports interaction network, RNA dynamic expression and four RNA interactome tools: RIscoper (23), IntaRNA (24), PRIdictor (25) and DeepBind (26) (Figures 1 and 4). In total, RNAInter integrated >41 million RNA-associated interactions across 154 species. It will provide a valuable resource for better understanding the RNA interactome.

**Table 1.** Overview of curated interaction data from 35 resources

Evidence type	Interaction type	Interaction entry	Database resource	Reference
Experimental validation	RCI	4525	SM2miR	(27)
		4113	ncDR	(28)
		822	EmDL	(29)
	RDI	138 062	LnChrom	(30)
	RPI	1 530 693	POSTAR2	(31)
		199 835	TransmiR v2.0	(32)
	RRI	258 818	RISE	(33)
		155 622	LncRNA2Target v2.0	(34)
		7904	VIRmiRNA	(35)
		3028	LncACTdb 2.0	(36)
		2680	NPInter v3.0	(37)
		1846	OncomiRDB	(38)
		1213	ncRDeathDB	(39)
		559	miR2Disease	(40)
		405	sRNATarBase 3.0	(41)
		81	MNDR v2.0	(42)
		60	LncRNADisease 2.0	(43)
	RHI/RPI	9 515 123	ChIPBase v2.0	(44)
	RPI/RRI	1 246 631	starBase v2.0	(45)
		737 835	miRTarBase	(46)
Computational prediction	RPI	23 304 537	RNAct	(47)
	RRI	1 956 709	miRDB	(48)
		1 557 635	miRanda	(49)
		547 003	piRTarBase	(50)
		247 731	RepTar	(51)
		191 123	TargetScan	(52)
		149 817	EIMMo	(53)
		106 471	DroID	(54)
		74 884	ZIKV - CDB	(55)
		243	HumanViCe	(56)
		14	miRcode	(57)
Experimental validation/	RRI	538 529	VmiReg	(58)
Computational prediction	RPI/RRI	5 272 396	RAID v2.0	(22)
		327 123	RAIN	(59)
		110 293	ViRBase	(60)

## DATA ORGANIZATION

### Data collection

RNAInter integrated experimentally validated and computationally predicted RNA interactome data from the literature and another 35 resources (Table 1). Literature within PubMed (mainly from 2016 to 2019) was screened with the following keyword combinations: (RNA molecule) AND (other molecule) AND (interaction). The keyword in brackets represents (i) RNA molecule: RNA symbols or RNA category names and (ii) other molecule: RNA symbols or RNA category names, protein symbols or ‘transcription factor’ or ‘RNA-binding protein’ or ‘protein’, gene symbols or ‘chromosome’, compound symbols or ‘compound’ or ‘drug’, or histone modification symbols or ‘histone modification’; and (iii) interaction: ‘bind’ or ‘interact’ or ‘regular’ or ‘target’. Finally, we reviewed over 31 000 published studies that included 419 522 RNA-associated interactions. Diverse RNA-associated interactions were also integrated from 24 experimentally validated databases and 14 computationally predicted databases (22,27–60) (see details in Table 1).

To facilitate elucidating the role of RNA in molecular interactions, more annotation information for the interactors was collected, including RNA modification sites from RMBase v2.0 (61), RNA subcellular localization from RNALocate (62), and RNA editing sites from RADAR (63), DARNED (64) and Lncediting (65). Simultaneously, the transcript and protein sequences from Refseq (66) and miRBase (67) were included to visualize the structure of RNA and represent target sites by miRanda, RIsearch (68) (tools for predicting RRIs), or PRIdictor (tool for predicting RPIs). The experimentally verified RNA-binding sites in proteins documented in the RBPDB (69), RsiteDB (70) and PDB (71) databases were also incorporated. Furthermore, we integrated the orthology/paralogy gene sets from miRBase and NCBI Gene (72) to reveal the conservation of homologous RNA-associated interactions across species.

### Data procession

Integrating multisource data requires unifying them into common reference databases to annotate various interactors. Four major types of interactor symbols were used: (i) miRNA symbols from the miRBase database, (ii) DNA, RNA and protein symbols from the NCBI Gene or Ensembl (73) database, (iii) compound symbols from the PubChem Compound (74) database and (iv) histone modification symbols from the ChIPBase v2.0 database. Notably, each histone undergoes various modifications, and we separated RNA–histone modification interactions (RHIs) from RPIs to specify the relationship between RNA and histone modification. Additionally, Entrez ID, Ensembl Gene ID, miRBase accession, PubChem Compound CID and their external links are also provided, which can efficiently retrieve a substantial amount of genome-associated information from external resources. For the convenience of users, interactor information also included NCBI Aliases, DrugBank Aliases, OMIM ID, HGNC ID, HPRD ID, UniprotKB protein accession, among others. The software ‘RNAstructure’ (75) was used to predict RNA secondary structure.

In particular, we collected and processed four single-cell RNA-seq (scRNA-seq) data sets from the Gene Expression Omnibus (GEO) (76) to visualize the RNA molecular dynamic expression pattern during diverse stages of human (or mouse) spermatogenesis and HSC lineage commitment (77,78). Firstly, scRNA-seq reads were downloaded and processed to remove adaptor contaminants and low-quality bases using trimmomatics v0.36 (79). The processed clean reads were aligned to the human and mouse reference genome (hg38 and mm10 from GENCODE) using TopHat v2.0.12 (80). The HTSeq v0.11.0 (81) was used to estimate the gene expression of each single-cell. The transcript copy number, counted by distinct unique molecular identifiers (UMIs), was obtained by removing duplicated transcripts according to the UMI information. For a given cell, the number of UMIs represents the transcript number of each gene. Secondly, we filtered out cells with fewer than 2000 genes and 10 000 transcripts to retain high-quality cells. In total, we obtained 2414 human bone marrow cells (GSE75478), 99 mouse precursor-haematopoietic stem cells (GSE67120), 2,435 human testicular cells (GSE106487) and 1136 mouse spermatogenic cells (GSE107644). The RNA expression levels were normalized by transcripts per million (TPM). Finally, we evaluated the correlation between two RNAs with the Pearson correlation coefficient (PCC) during human (or mouse) spermatogenesis and HSC lineage commitment.

## RESULTS

### RNAInter statistics

**Figure 2.**
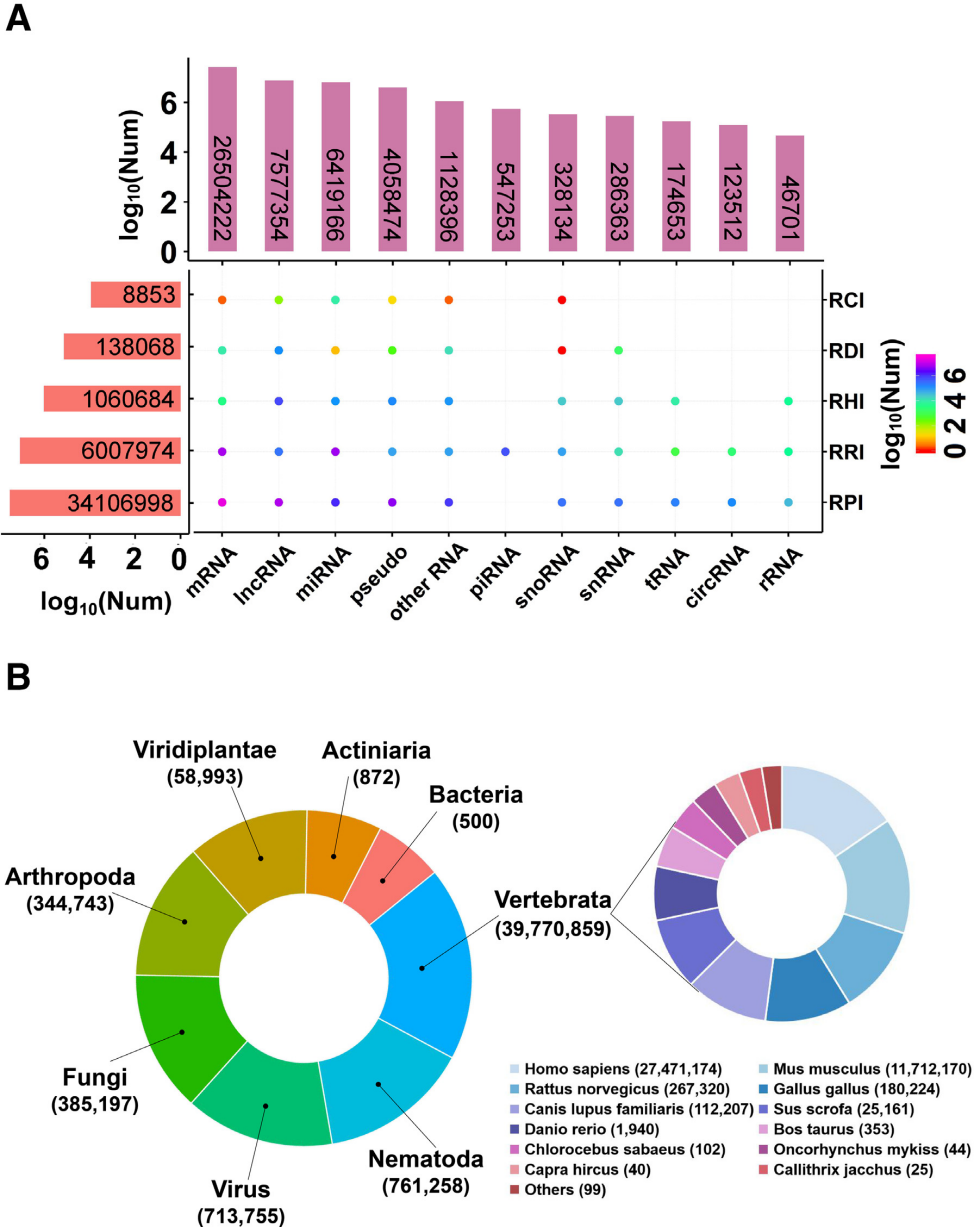
Statistics on RNAInter. (**A**) The distribution of five interaction types (RCI/RDI/RHI/RPI/RRI) in 22 RNA categories. The category ‘other RNA’ includes eRNA, ncRNA, others, repeats, ribozyme, scaRNA, scRNA, shRNA, sncRNA sRNA, unassigned RNA and unknown. (**B**) Number of interactions in vertebrata, nematoda, virus, arthropoda, fungi, viridiplantae, actiniaria, bacteria (left) and 28 species belonging to vertebrata (right).

In summary, RNAInter contains 41 322 577 RNA-associated interactions, including 34 106 998 RPIs, 6 007 974 RRIs, 1 060 684 RHIs, 138 068 RDIs and 8853 RNA-compound interactions (RCIs) (Figure 2A, Table 1). These interactions involve 381 319 nonredundant RNAs and 42 215 nonredundant proteins, 33 970 newly added nonredundant DNAs, 425 nonredundant compounds and 61 nonredundant histone modifications. RNAInter involved 22 RNA types, eight of which added for the first time, including enhancer RNA (eRNA), Piwi-interacting RNA (piRNA), repeats, ribozyme, short hairpin RNA (shRNA), small Cajal body-specific RNA (scaRNA), small RNA (sRNA) and noncoding RNA (indefinite classified ncRNA) (Table 2). The distribution of the five types of interactions among different RNAs is shown in Figure 2A. The number of organisms in RNAInter increased from 60 to 154 compared with that in RAID v2.0 (Table 2). All the species covered nine categories (actiniaria, arthropoda, bacteria, fungi, mycetozoa, nematode, vertebrata, viridiplantae, virus). *Homo sapiens* and *Mus musculus* interactions took up the main part of the vertebrata (Figure 2B). Other model organisms, such as *Drosophila melanogaster, Rattus norvegicus, Saccharomyces cerevisiae* and zebrafish (*Danio rerio*), have also been documented in RNAInter.

**Table 2.** The features and developments of RNAInter

Feature	RAID v1.0	RAID v2.0	RNAInter
Interaction entry*	6112 (6112)	5 272 396 (2 426 181)	41 322 577 (13,653,108)
RNA symbol	2070	118 878	381 319
Species coverage	1	60	154
Interaction type	RNA-protein/RNA-RNA	RNA-Protein/RNA-RNA	RNA-Protein/RNA-RNA/RNA-Compound/RNA-DNA/RNA-Histone modification
RNA category	lncRNA/miRNA/mRNA/rRNA/snoRNA	circRNA/lncRNA/miRNA/miscRNA/mRNA/pseudogene/rRNA/scRNA/sncRNA/snoRNA/snRNA/tRNA	circRNA/lncRNA/miRNA/miscRNA/mRNA/pseudogene/rRNA/scRNA/sncRNA/snoRNA/snRNA/sRNA/tRNA/eRNA/ncRNA/piRNA/repeats/ribozyme/scaRNA/shRNA/sRNA
Detailed information	Basic annotations/Evidence support/Reference/Tissue or cell line	Basic annotations/Evidence support/Interactor homolog/Integrated confidence score/Reference/RNA-binding sites	Basic annotations/Evidence support/Interactor homolog/Integrated confidence score/Reference/RNA-binding sites/Homology interaction/RNA editing/RNA localization/RNA modification/Target region
Data visualization	Predicted binding sites/ Interaction network	Predicted binding sites	Predicted binding sites/Interaction network/RNA dynamic expression/RNA structure
Web application	-	Advanced filter search	Exact search/Batch search/Fuzzy search/Four interactome tools: RIscoper, IntaRNA, PRIdictor, DeepBind

*The number in brackets counts interactions entries verified by experimental methods.

### Data feature and utility

Then, we expanded the RNA-compound, RNA–DNA and RNA–histone modification interactions in RNAInter. Apart from basic annotation, support evidence, RNA-binding sites and references, we focused on the multifaceted supplementation of the details of RNA editing/localization/modification/structure/dynamic expression, the interaction network, the target region and the homology interaction in detail. ‘RNA editing’ provides editing position, editing type and genetic region. ‘RNA localization’ includes subcellular localization and the tissue or cell line. ‘RNA modification’ involves the modification position, modification type and genetic region. Moreover, ‘Homology interaction’ shows the conservative interactions across organisms documented in RNAInter. ‘Target region’ shows the target locus in RHI/RPI/RRI and data accession from the literature or high-throughput sequencing with their sample resources. All this information links to their corresponding databases.

**Figure 3.**
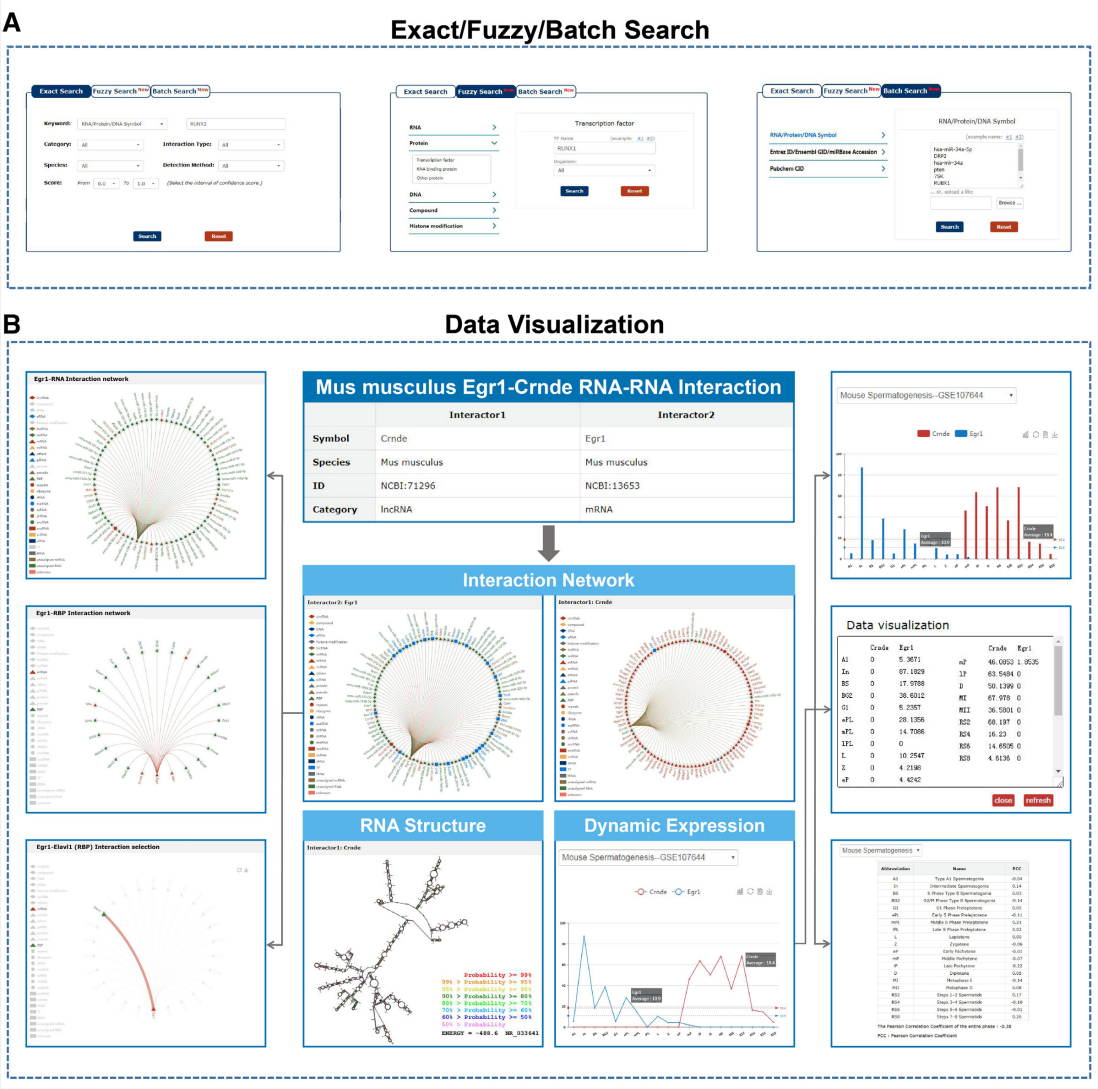
New search function and data visualization of the RNA interactome. (**A**) Presentation of exact, fuzzy and batch search described in the search options. (**B**) Visualization of the interaction network, RNA structure and RNA dynamic expression.

RNAInter provides a user-friendly platform for searching, browsing, visualizing and profiling RNA interactome data. To improve the search capability, RNAInter enables an optimized query with a new function of fuzzy and batch search. Fuzzy Search can help users to search interactions using unstandardized or uncertained interactor name under selected molecular category, then the result of interactions will be presented by selecting interactors in candidate list. Meanwhile, Batch Search supports for inputting a list of official symbols/IDs or uploading a file with text format to obtain multiple molecular categories associated interactions. Thus, users can select ‘Exact Search’ to filter the search results, or ‘Fuzzy Search’ to further focus on interactors of interest, or ‘Batch Search’ to customize their query content in batch (Figure 3A). Taking the load time into account, RNAInter offers the download option for over 2 million entries on the ‘Browse’ page. ‘RNA structure’ represents the putative RNA secondary structure for each transcript. In addition, ‘Interaction network’ is offered to picture the top 100 interactions ranked by integrative confidence score in RNAInter. Users can also select specific categories of RNA-associated interactions by clicking the different icons of interactor to conceal uninterested interactions for superior view. Click any edge of the network can jump to a detailed page of the corresponding entry (Figure 3B). To illustrate the RNA molecular dynamic expression pattern, ‘Dynamic expression’ shows the line chart of RNA expression values in each stage during human (or mouse) spermatogenesis and HSC lineage commitment and their expression correlation in each stage and entire phage with PCC (Figure 3B). The images of the interaction network and dynamic expression pattern can be downloaded.

### Extended toolkit

**Figure 4.**
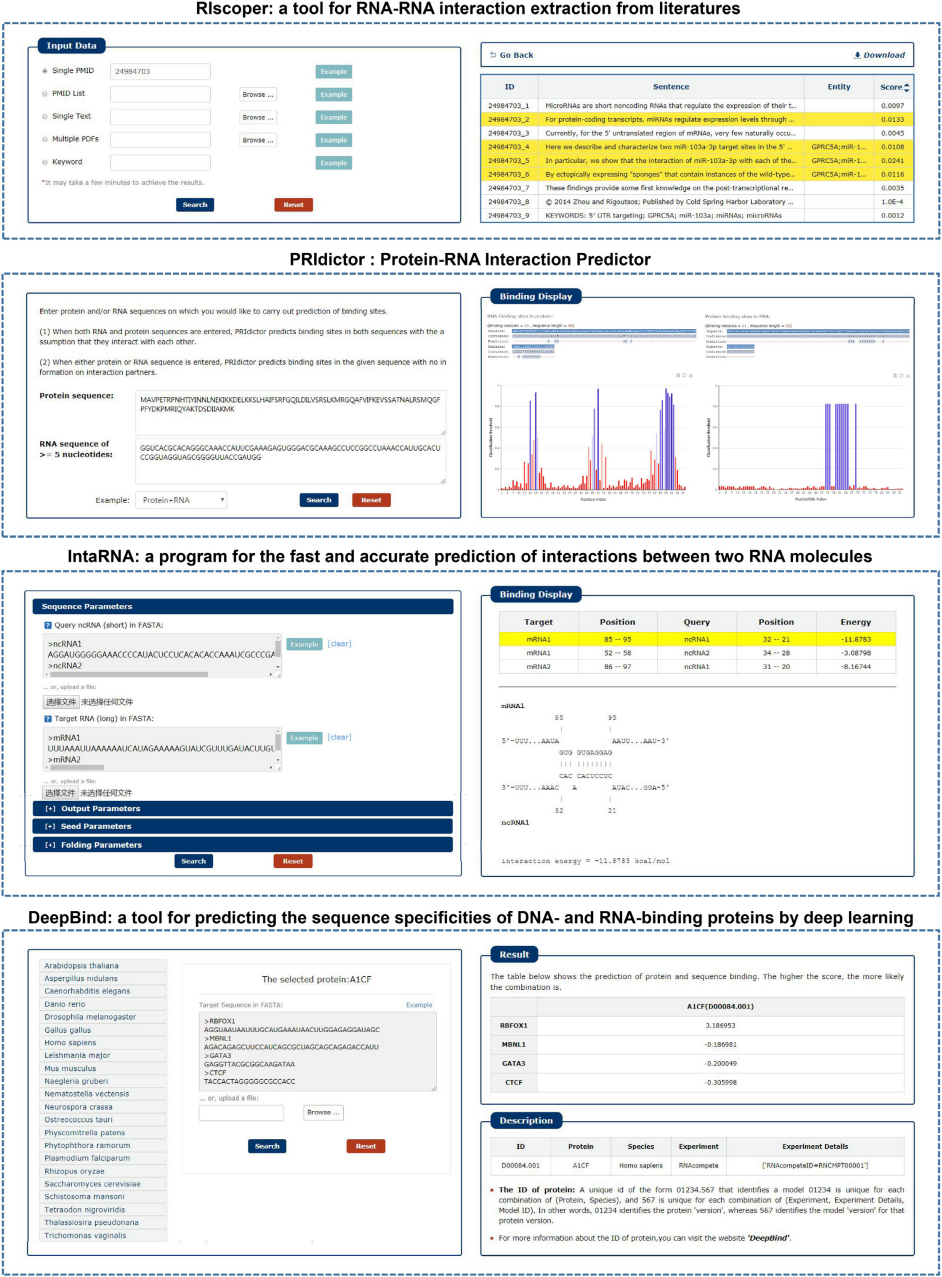
Snapshot of four RNA interactome tools in RNAInter: RIscoper, PRIdictor, IntaRNA and DeepBind (left: input option, right: result presentation).

In response to the diverse needs of users, RNAInter embeds four interactome tools: RIscoper, IntaRNA, PRIdictor and DeepBind. RIscoper is a tool for RNA–RNA interaction extraction from the literature. IntaRNA is a program for the fast and accurate prediction of interactions between two RNA molecules. PRIdictor is a protein–RNA interaction predictor. DeepBind predicted the sequence specificities of DNA- and RNA-binding proteins by deep learning (Figure 4).

### CONCLUSIONS AND PERSPECTIVES

RNAInter is an update of RAID v2.0, a comprehensive resource for RNA interactome data obtained from the literature and other databases, containing over 41 million RNA-associated interactions of RCI, RDI, RHI, RPI and RRI. With detailed interactome information, visualized interaction network and RNA dynamic expression, enhanced search functions, and embedded RNA interactome tools, RNAInter depicts a system-level RNA interactome landscape with guides and help researchers to perform further studies. We expect RNAInter to update the manual curation of RNA interactome data and expand the available information about RNAs and other molecules in the future. Continuously integrating high-throughput data, including scRNA-seq, to provide more precise depiction of the dynamic expression pattern of RNAs illuminates the role of RNA across organisms. We may optimize the confidence score strategy with the emergence of new mass sequencing technologies, experimental methods and prediction algorithms. At the same time, more RNA-associated applications are docking with our database. Eventually, RNAInter will present the most comprehensive map of the RNA interactome to satisfy different requirements.

## Supplementary Material

gkz804_Supplemental_File
