# Supplementary material for: RNAInter in 2020: RNA interactome repository with increased coverage and annotation
Source: Nucleic Acids Res. 2019 Sep 17;48(D1):D189–97. doi: 10.1093/nar/gkz804 (PMC6943043; doi:10.1093/nar/gkz804)
Supplement: gkz804_Supplemental_File [file gkz804_supplemental_file.pdf]

Supplementary Table1. The brief introduction of RNA interactome detection techniques.

**Abbreviation**	**Full name**	**PMID**	**Description**
Degradome-seq*	Degradome sequencing	18472421	Degradome-seq is a transcriptome-wide experimental method, directly detect cleaved miRNA targets without relying on predictions or over-expression. Degradome sequencing has been applied to identify miRNA-directed mRNA cleavage and understand the biological function of miRNAs and their target genes in plants defense to stress.
LIGR-seq*	LIGation of interacting RNA followed by high-throughput sequencing	27184080	LIGR-seq is a rapid and versatile method for the global-scale detection of RNA-RNA interactions in vivo that does not require prior knowledge of RNAs forming interactions, or of proteins required for such interactions.
MARIO*	Mapping RNA interactome in vivo	27338251	MARIO technology identified RNA–RNA interactome is composed of tens of thousands of interactions, which involve mRNA, lincRNA, snoRNA, small nuclear RNA, tRNA, miRNA, transposon RNA, pseudogene RNA, antisense RNA and novel transcripts. And this technology detect RNA–RNA interactions facilitated by any single protein in vivo.
PARIS*	Psoralen Analysis of RNA Interactions and Structures	27180905	PARIS, a method based on reversible psoralen crosslinking for global mapping of RNA duplexes with near base-pair resolution in living cells. It determines base-pairing interactions on an individual-molecule level, revealing pervasive alternative conformations.
dCLIP†	denaturation step to UV-crosslink RNA immunoprecipitation	29073373	Denaturing CLIP (dCLIP), pipeline for identifying discrete RNA footprints on chromatin-associated proteins, which nables purification under denaturing conditions to maximize binding specificity. dCLIP yields small RNA footprints of 150–200 nucleotides on average, thereby enabling deduction of four families of motifs for CBX7 binding.
PAR-CLIP†	Photoactivatable-Ribonucleoside-Enhanced Crosslinking and Immunoprecipitation	20371350	PAR-CLIP: transcriptome-wide identification of RNA-binding protein and microRNA target sites.

RIP-seq†	RNA immunoprecipitation followed by high-throughput sequencing	21172659	RIP-seq applies native RNA immunoprecipitation (RIP) with a RBP of interest in cell lysates and is followed by RNA-seq.
uvCLAP†	ultraviolet crosslinking and affinity purification	29559621	uvCLAP, an easy-to-use, robust, reproducible, and high-throughput method to determine in vivo targets of RBPs. uvCLAP is fast and does not rely on radioactive labeling of RNA.
ChIRP-seq‡	Chromatin isolation by RNA purification sequencing	21963238	ChIRP-seq is generally applicable to illuminate the intersection of RNA and chromatin with new found precision genome-wide.
ChOP-seq‡	Chromatin oligo affinity precipitation sequencing	25584904	Deep sequencing of the ChOP DNA showed that RNA occupies thousands of loci on the chromatin.
diMARGI‡	direct Mapping RNA-genome interactions	28132817	diMARGI is designed to reveal protein or RNA tethered interactions, which is a variation of the MARGI (mapping RNA-genome interactions), a technology to massively reveal native RNA-chromatin interactions from unperturbed cells.
GRO-seq‡	Global run-on sequencing	23945587	GRO-seq, an assay that allows mapping and quantification of transcriptionally engaged RNA polymerases and provides a snapshot of genome-wide transcription. It is the most widely used method to measure nascent RNA, and in recent years, it has been applied successfully to study the function and mechanism of action of noncoding RNAs.

*This technique uses to detect RRIs.

†This technique uses to detect RPIs.

‡This technique uses to detect RDIs.
